# First report of Culex flavivirus infection from *Culex coronator* (Diptera: Culicidae), Colombia

**DOI:** 10.1186/s12985-018-1108-2

**Published:** 2019-01-03

**Authors:** Jorge Miranda, Salim Mattar, Marco Gonzalez, Richard Hoyos-López, Ader Aleman, Jose Aponte

**Affiliations:** 1 Instituto de Investigaciones Biológicas del Trópico, Facultad de Medicina Veterinaria y Zootecnia, Universidad de Córdoba, Montería, Colombia; 2 Grupo de Investigación en Enfermedades Tropicales y Resistencia Bacteriana, Universidad del Sinú, Montería, Colombia

**Keywords:** Arboviruses, *Culicidae*, Environment and public health, Insect vector, Flaviviruses, Tropical climate

## Abstract

**Background:**

Flaviviruses are important pathogens for humans and animals (Dengue viruses, Yellow fever virus, Zika virus and West Nile virus). Culex flavivirus (CxFV) is an insect-specific virus of the genus *Flavivirus,* detected in a wide variety of mosquito species.

**Objective:**

To detect Flavivirus in mosquitoes of a tropical region of the Colombian Caribbean.

**Methods:**

In 2014, an entomological surveillance of arboviruses was conducted in the department of Cordoba area of the Caribbean, Colombia. A total of 8270 mosquitoes were captured as follow: *Mansonia* (*n* = 3271/39.5%), *Culex* (*n* = 2668/32.26%), *Anopheles* (*n* = 840/10.15%), *Aedeomyia* (*n* = 411/4.9%), *Psorophora* (*n* = 397/4.8%), *Coquilletidia* (*n* = 369/4.46%), *Uranotaenia* (*n* = 261/3.15%) and *Aedes* (*n* = 53/0.6%). All mosquito species were collected in dry tropical forest of the Caribbean area. Universal primers for NS5 gene (958 pb), RT-PCR for flavivirus and sequencing were used for molecular identification of viruses detected.

**Results:**

Two pools belonging to *Culex coronator* were positive for flavivirus RNA sequence by RT-PCR. The sequences of the PCR amplicons, matched that of the Culex flaviviruses, CxFv COL PM_149 (GenBank: KR014201) and CxFv COL PM_212 (GenBank: KT307717). Phylogenetic analysis of the NS5 protein sequences of the Culex flaviviruses sequences with those of reference sequences available in GenBank indicated viruses of Genotype II, closely related to the Brazilian strain, BR_SJRP_01_ (GenBank: KT726939), from *Culex* sp. The alignment of Culex flavivirus sequences CxFv COL_ PM 212 and CxFv COL_ PM 149 with sequences of strains detected in different geographical regions grouped the strains in a Latin American clade reported in Brazil, Argentina and Mexico.

**Conclusions:**

The present work illustrated that CxFV was circulating among vectors of human pathogenic arboviruses in Colombia, but the impact of CxFV on other flaviviruses which are endemic in the study area still remains to be explored.

**Electronic supplementary material:**

The online version of this article (10.1186/s12985-018-1108-2) contains supplementary material, which is available to authorized users.

Culex flavivirus (CxFV) is an insect-specific virus of the genus Flavivirus detected in mosquitoes. The present report shows the first detection of CxFV in mosquitoes *Culex coronator* in Colombia. The strains detected are phylogenetically related to Africa/Caribbean/Latin America genotype 2.

## Background

Arboviruses are a serious public health problem in tropical countries, the genus flavivirus included a group of these viruses which some of them are highly pathogenic, and can cause encephalitis or hemorrhagic fevers in humans [1]. In each region, there are different mosquito species vectors and different animals which serves as virus reservoirs.

Culex flavivirus (CxFV) is an insect-specific virus of the genus Flavivirus. CxFV strains have been detected from mosquitoes *Culex pipiens, Culex quinquefasciatus*, and other *Culex* sp. around the world [2, 3]. CxFV belongs to *Flaviviridae,* the viruses within this family are transmitted horizontally between vertebrate hosts and hematophagous arthropods. However, certain members of the genus Flavivirus are known to be vertebrate-specific whilst others are insect-specific flaviviruses (ISFV). Transmission of ISFVs in natural mosquito populations is maintained by vertical transmission and in minor role the venereal transmission [4–6].

In Europe, species of ISFV have been identified in field mosquitoes from Italy, Portugal, Spain, the United Kingdom, the Czech Republic and Greece [7–9]. Sequences related to those viruses have been detected worldwide. In Algeria, Spain and Portugal ISFV RNA has also been detected in sand flies of the family *Psychodidae* [8]. Despite the large number of ISFV, they have been not completely characterized phylogenetically [8].

ISFV do not cause disease in humans or animals, but they are important because they co-infect mosquito vectors and compete with the viruses that are pathogenic to humans and animals [10].

The circulation of ISFVs in nature is of increasing interest due to their influence onarboviruses transmission [9]. In vitro co-infection studies with mosquito-borne flaviviruses and ISFV have shown that they alter vector competence for the mosquito-borne flaviviruses in both enzootic and epizootic transmission cycles [10]. Several investigations have addressed co-infection exclusion effects between CxFV and arboviruses such as West Nile virus (WNV) and Rift Valley fever virus, however, studies are not conclusive [10]. Ongoing questions about the role of ISFV in the control of pathogenic arboviruses like dengue, Yellow Fever (YF), Zika and Ckinkungunya suggest the possibility of their use in the biological control of arbovirus vectors.

Colombia, due to its geographical location, has a great biodiversity of reservoirs and arthropods, which have supported the emergence of viruses such as Chikungunya and Zika. These viruses have adapted to the vectors circulating in the tropics, which facilitated their spread, and caused the two most recent major epidemics of arboviruses in the Americas. The Colombian Caribbean also maintains circulation of pathogenic Saint Louis encephalitis virus (SLEV) and West Nile Virus (WNV), transmitted by mosquitoes of *Culex erraticus, Cx. quinquefasciatus* and *Mansonia titillans*, confirming that these ecological conditions are favorable to high viral biodiversity [11–13].

The objective of this work was to detect Flaviviruses in mosquitoes of a tropical region of the Colombian Caribbean.

## Methods

### Type of study, geographic area and sampling sites

From August 2012 to December 2014, an observational, descriptive and prospective study was conducted to identify circulating flaviviruses in different mosquito species of the department of Córdoba, Colombia. The department is located in the Caribbean region between 09° 26′16" and 07° 22′05 “N, and 74° 47’ 43” and 76° 30’01 ‘W. The department of Córdoba has areas with wetlands and tropical dry forest, the area is characterized by rainfall that does not exceed 2000 mm per year, of which, 80% is during the rainy season (May-October). The tropical climate has an average annual temperature of 28°C, with heights between 0 and 40 masl. The department is divided geographically into sub-regions according to the territorial development plan: High, Medium and Low Sinú, and the samplings were carried out in these areas. One of the geographical zones corresponded to the municipality of Montería (8 ° 43’15.80 “N -075 ° 59′27.27” W (Medium Sinú), where the circulation of WNV and SLEV have been reported [14, 15]. The two remaining areas were the municipalities of Tierralta (8° 09′19.20 “N -076° 00′ 55.40” W-High Sinu) and Purisima (9° 14’ 57.60 “N -075° 42′ 34.62” W -Low Sinú)

### Mosquitoes collections

In each of the selected sites, a total of five samplings were set-up. Four Light-CDC traps were used to capture mosquitoes, and 2 BG Centinell ™ traps fed with CO^2^ were used to capture gravid females of different genera. The sampling sites were close to small lagoons and areas of tropical dry forest. During each sampling, the traps were placed between 17:00 and 6:00 h. Manual aspirators were also used. The collected mosquitoes were kept at 6 °C, until being transported to the laboratory of the Biological Research Institute of the Tropic (IIBT), in less than 12 h post-capture. Once in the laboratory the mosquitoes were stored at − 80 °C for molecular analysis.

### Taxonomic identification of mosquitoes

Male mosquitoes were identified first. It was necessary to clarify Terminalia [16], due to the difficulty to identify some species of the *Culex* genus. They were classified according to their morphology based on specialized taxonomic keys [16–18]. To morphological confirmation and mounting of genitalia, it was verified the characters used to taxonomical identification such as: 1. Subapical lobe of the gonocoxite as a quadrate at the outer third, bearing a row of eight rods that are progressively smaller distally; gonostyle bearing a small terminal claw and two minute setae situated on inner face before tip, ventral arm of the phallosome long and curved, and lateral arm with several large teeth with angular rounded corners, using description provided by Howard (1915). Recently discussion about this species by Laurito et al. (2017), allow consider other description about genitalia of male, such as: subapical lobe of the gonocoxite more or less divided with four similar stout rod-like, setae on the proximal part of the lobe (toward the base of the gonocoxite) and several subequal filiform setae on the distal part of the lobe (toward the apex of the gonocoxite); gonocoxite with an apical cluster of fine setae that extend to mid-length of the gonostylus [19]. For females we used Forattini characters described as: 1. Feminine maxillary palps are entirely dark. 2. There are no post-spiracular scales in the pleura. 3. The wing and devoid of sets of clear scales. 4. The tarsi are clearly marked and the tarsomer 5 presents a clear basal area [20]. The identification was carried out at 14 °C. Pools of 2 to 50 individuals of engorged or non-engorged female mosquitoes of the same species, location area, date and type of trap were grouped.

### Flavivirus detection

RNA extraction was performed using the QIAamp Viral RNA Mini kit (QIAGEN, Inc.) following the manufacturer’s instructions. The RNA was extracted from 140 μl of the homogenized mosquitoes sample. For detection of flavivirus reverse transcription RT-PCR with universal primers previously described by Bronzoni et al. [21] were used; the primers amplify a fragment of 958 base pairs (bp) of the NS5 protein: Forward FG1-TCAAGGAACTCCACACATGAGATGTACT and Reverse FG2-GTGTCCCATCCTGCTGTGTCATCAGCATACA. The positive control used was Dengue virus 2.

### Sequencing and phylogenetic analysis

The amplified fragments were sequenced in both directions in an ABI 3730 XL Automated sequencer. Thirty-six representative sequences belonging to different geographic regions were downloaded from Genbank and aligned using MUSCLE in MEGA v7 [22]. Alignments in FASTA format were also evaluated in jModelTest v2.1.4 [23], using the Akaike criterion informative to model appropriate nucleotide substitutions (Additional file 1). The XML file for bayesian analysis was built in BEAUti v1.5.4 http://beast.bio.ed.ac.uk/Main_Page, to model sequence evolution, invariants, gamma distribution, size of the chain run (20 million generations), coalescent constant population and uncorrelated molecular clock were chosen for this inference [24].

## Results

**Fig. 1:**
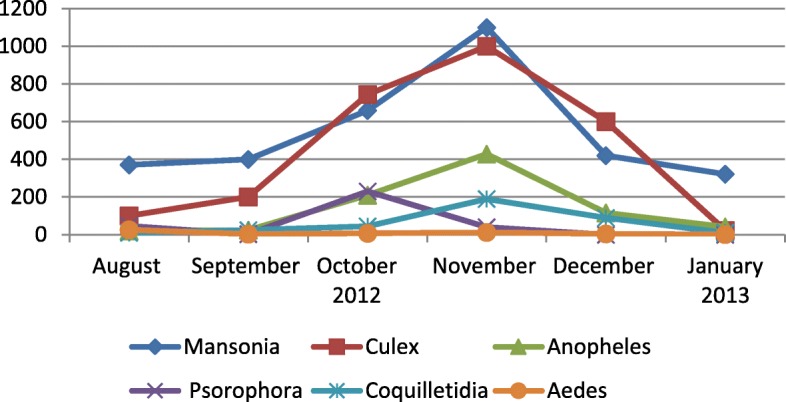
Distribution monthly of mosquitoes of great medical importance captured in the study

Nine thousand eight hundred ninety eight mosquitoes were captured, and 8270 of them were identified and grouped in 334 pools. 41 species were identified and distributed in 8 genera: *Mansonia* (*N* = 3271/39.5%), *Culex* (*n* = 2668/32.2%), *Anopheles* (*n* = 840/10.1%), *Aedeomyia* (*n* = 411/4.9%), *Psorophora* (*n* = 397/4.8%), *Coquilletidia* (*n* = 369 / 4.4%), *Uranotaenia* (*n* = 261/3.1%) and *Aedes* (*n* = 53/0.6%). The genus of mosquitoes of greater medical importance, distributed monthly is shown in the Fig. 1.

### Detection of viral RNA in mosquitoes and sequencing analysis

Of the 334 analyzed pools, 2 pools of *Culex coronator* were positive for flavivirus. These mosquitoes were collected in the municipality of Montería (PM 149 and PM 212). The 958-bp amplicons from the NS5 gene were sequenced, and the sequences were edited and assembled to obtain a fragment of 758 bp for analyses. Sequences were aligned and a 99% similarity was obtained with *Culex flavivirus* virus sequences from position 8142 to position 8899 of the complete genome of the isolate (Strain: BR_SJRP_01_2012, Genbank accession number: KT726939). Sequences were deposited in the GenBank: CxFv COL_ PM 212 (Genbank accession number: KT307717) and CxFv COL_ PM 149 (Genbank accession number: KR014201).

### Phylogenetic analysis

**Fig. 2:**
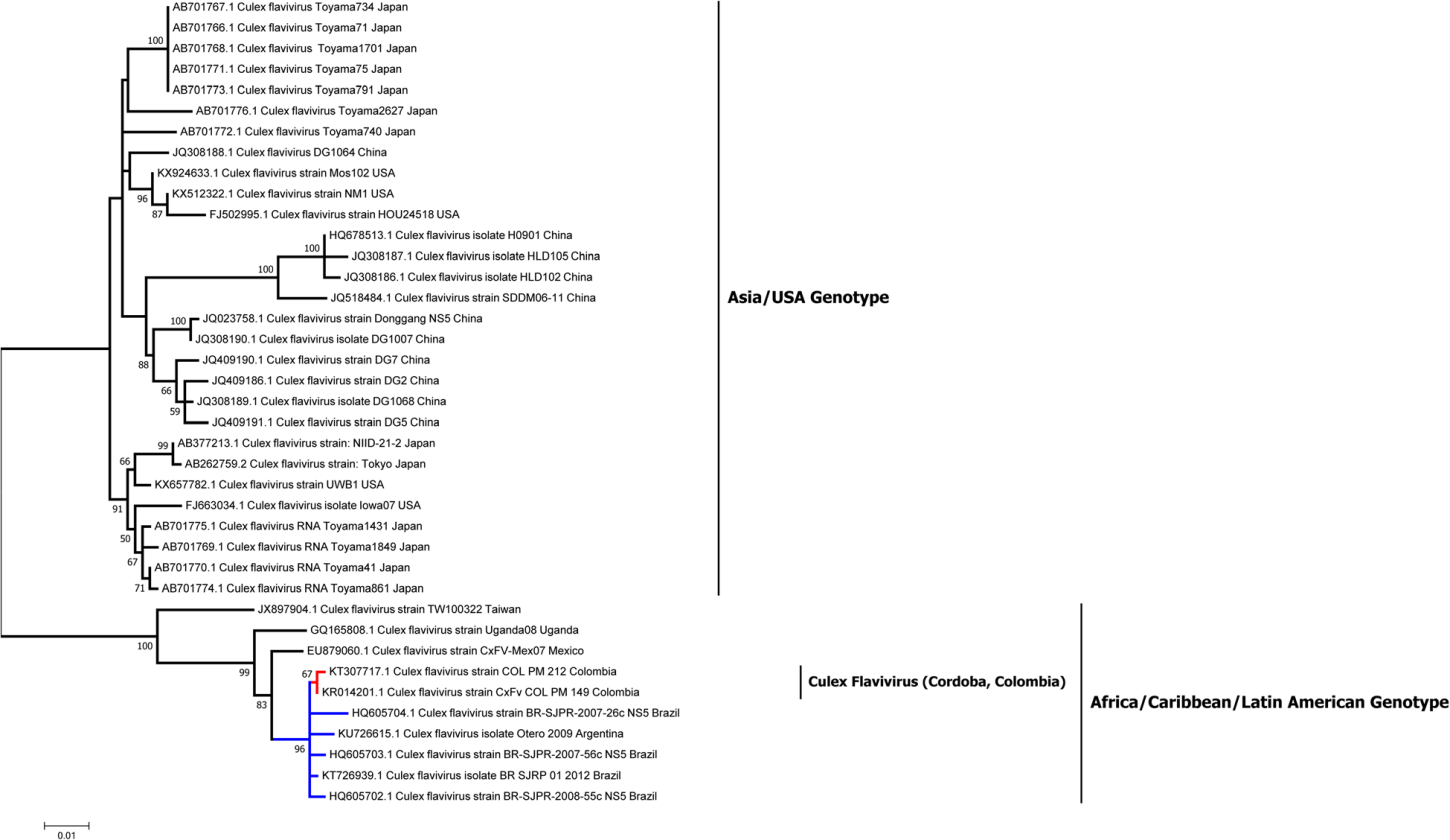
Phylogenetic tree estimated by Bayesian analysis of 80 sequence strains (NS5) of Culex Flavivirus virus data under the GTR + G + I model of nucleotide substitution. The number accession of Genbank is followed by abbreviated name of strain. The branches in red belong to our characterized sequences

Alignments of the NS5 sequences from the *Culex* flaviviruses, CxFv COL_ PM 212 and CxFv COL_ PM 149, with NS5 sequences from strains detected in different geographical regions allowed their grouping in a clade that includes virus strains reported in Brazil, Argentina and Mexico and belonging to the genotype II group of strains of Africa/Caribbean/Latin America (Fig. 2).

## Discussion

In the present study, the most abundant mosquito genera were *Mansonia* (39.5%) and *Culex* (32%), these results differ somewhat from those reported by Hoyos et al. [12] in a nearby area where the genus *Culex* (50%) was the most abundant. The difference between these results is possibly due to the geographic, climatic and environmental characteristics of the selected areas.

The limited number of positive samples (pools) in the present study 0.6% (2/334 pools) is similar to reported by Bryant et al 2005 in Vietnam, where only 2.3% of analyzed pools were positives (26/1122) [25]. Ochieng et al., during 2007 and 2012 in Kenia, captured 450.680 mosquitoes and analyzed 15.890 groups, 53 were positives 0.3% [26]. Loftin et al., in Virginia, USA, reported 0.8% of positives pools of 14.949 analyzed [27]. Finally, Burgueño in Uruguay analyzed 244 pools and found only 2% (5 pools) of positives pools with Culex Flavivirus virus. However, Morais-Bronzoni et al., suggest that nested PCR assays for detection of flaviviruses strategy could be more sensitive in samples with low viral load [21].

According to the taxonomic definition of flaviviruses, based on the identity of the conserved region of NS5 gene (> 84%) [28], the detected strains CxFv COL_PM 212 and CxFv COL_PM 149 are specific ISFV of the species CxFV. The sequences detected in the present study have an identity of 99% (756/758 bp) with isolated CxFV BR_SJRP_01_2012, (KT726939) from Brazil [29]. These results confirm for the first time the presence of CxFV in *Cx. coronator* species in Colombia. CxFV was previously reported in Colombia, in the department of Córdoba in *Cx. quinquefasciatus* (KM031075) and *Cx. erraticus* (KM031073) species [12]. Interestingly, the nucleotide identity between the CxFV strains reported by Hoyos et al., was 94% (338/358 bp) with respect to ours results, which implied a possible wide range of strains with significant genetic diversity.

CxFV have a great diversity of reservoirs and their phylogeny could be influenced by these. The phylogenetic tree shows two clades (Fig. 2): the first clade is associated with Asian strains and strains from the USA, where *Cx. pipiens* is the main reservoir. However, other mosquito species, such as *Cx. tritaeniorhynchus* and *Anopheles sinensis* [30], have been reported with CxFV in the first clade. These isolates were classified in genotype I (Asian/USA).

The second phylogenetic group of CxFV have been reported mostly in the Americas and Africa, with *Cx. quinquefasciatus* as the main reservoir [3, 29, 30], the strains detected in this study are more phylogenetically related to this group, described as genotype II, (Africa/Caribbean/Latin America; Fig. 1). However, it is important to clarify that the genotypes are based on the phylogenetic analysis of the viral envelope protein (E) and our phylogenetic was performed with the NS5 gene.

The present study reports the first description of CxFV in *Cx. coronator* in the department of Córdoba, the presence of these viruses in mosquitoes could play an important role from the public health point of view, because it has been shown that previous CxFV infection can increase or block the infection of the mosquito by other pathogenic flaviviruses. CxFV-positive mosquitoes also differ in flight activity, which could decrease contact with arbovirus amplification hosts [10, 31–34]. Future studies should be carried out, in order to detect CxFV in other areas of Colombia to define the distribution of the virus, its genetic characteristics and relationship with other mosquito species.

## Additional file

Additional file 1: Culex flavivirus aligment Bayesian inference phylogeny of representatives CxFv sequences belonging to different geographic regions strains obtained from the GenBank database. (FAS 29 kb)

## Abbreviations

bp: base pairs
COL: Colombia
Cx: *Culex*
CxFV: Culex flavivirus
ISFV: insect-specific Flavivirus
NS: non-structural protein
RT-PCR: Reverse transcription polymerase chain reaction
SLEV: Saint Louis encephalitis virus
WNV: West Nile Virus
YF: Yellow Fever
